# Effects of a rehabilitation program on microvascular function of CHD patients assessed by near‐infrared spectroscopy

**DOI:** 10.14814/phy2.14145

**Published:** 2019-06-12

**Authors:** Rogerio N. Soares, Juan M. Murias, Flavia Saccone, Leopoldo Puga, Gustavo Moreno, Miguel Resnik, Gabriela F. De Roia

**Affiliations:** ^1^ University of Calgary Faculty of Kinesiology Calgary Alberta Canada; ^2^ Sanatorio Dr. Julio Mendez CABA Buenos Aires Argentina; ^3^ CENARD Centro Nacional de Alto Rendimiento Deportivo CABA Buenos Aires Argentina; ^4^ Universidad de Flores CABA Buenos Aires Argentina

**Keywords:** Cardiovascular disease, microvascular responsiveness, NIRS, rehabilitation, vascular

## Abstract

This study aimed to evaluate whether near‐infrared spectroscopy (NIRS)‐derived reperfusion slope would detect the effects of a 12‐week rehabilitation program on lower limb microvascular responsiveness in patients with coronary heart disease (CHD). Ten CHD patients (7 males and 3 females; 57.3 ± 7.6 years) underwent 12 weeks of drug treatment and high‐intensity interval training (HIIT), 2 times per week (40 min/session). Microvascular responsiveness was assessed by using NIRS assessment of muscle oxygen saturation (StO_2_) combined with a vascular occlusion test (VOT) (NIRS‐VOT). NIRS‐VOT measures were taken at pre‐ and postintervention, and microvascular responsiveness was evaluated by examining the slope 2 of re‐oxygenation rate (slope 2 StO_2_) and the area under the curve (StO_2_ _AUC_) of StO_2_ signal following cuff release subsequent to a 5‐min occlusion period. The slope 2 StO_2_ was significantly steeper after 12 weeks of training (4.8 ± 1.6% sec^−1^) compared to the pretraining (3.1 ± 1.6% sec^−1^) (*P* < 0.05). The area under the curve for the change in the % StO_2_ signal during re‐oxygenation increased significantly from 3494 ± 2372%∙sec at pretraining to 9006 ± 4311%∙sec at post‐training (*P* < 0.05). NIRS‐VOT technique detected the improvements of 12 weeks of rehabilitation program in the lower limb microvascular responsiveness of CHD patients.

## Introduction

Individuals with cardiovascular disease (CVD) show arterial stiffness, reduced skeletal muscle capillarization, and impaired ability of the peripheral arteries to dilate in response to an ischemia/reperfusion stress (i.e., impaired vascular responsiveness) (Zhang et al. 2000; Leung et al. 2008; Kalogeris et al. 2012). For example, a previous study using flow‐mediated dilation (FMD) to assess vascular responsiveness in patients suffering from coronary heart disease (CHD) showed that FMD was significantly reduced in this population and indicated vascular responsiveness as an independent predictor of long‐term cardiovascular events (Zhang et al. 2000).

Although pharmacological interventions improve vascular function in CVD patients, combining drug administration and exercise training programs are more effective interventions that also offer beneficial vascular effects in CVD patients and have become a primary objective of a cardiac rehabilitation program (Ayabe et al. 2004; Rakobowchuk et al. 2008; Tinken et al. 2008). Maiorana et al. showed that an 8‐week rehabilitation program that included medication for patient stabilization as well as whole‐body resistance and aerobic exercise training improved vascular reactivity in the radial artery of 12 patients with CVD (Maiorana et al. 2000). More specifically, it has been shown that exercise exerted beneficial effects on vascular responsiveness in healthy and patients with coronary heart disease, with higher intensities of training being even more effective than lower intensities of exercise in improving FMD and cardiovascular risk factors (Liuni et al. 2010; Ades et al. 2011).

Even though most of cardiovascular rehabilitation exercise interventions have shown beneficial effects on vascular responsiveness (Maiorana et al. 2000; Tinken et al. 2008; Guiraud et al. 2012), most of the studies investigating the effects of pharmacological and/or exercise intervention on vascular responsiveness in CVD populations have only assessed FMD in the forearm (Maiorana et al. 2000; Walsh et al. 2003; Currie et al. 2013). This is an important consideration as impairments in lower limb vasculature are also associated with morbidity and mortality in CVD patients (Dieter et al. 2002). Additionally, as assessing vascular function in the lower limb by using FMD is challenging and less reliable than upper limb measurements (McLay et al. 2016c), the majority of the studies evaluating the effects of exercise training interventions in the vasculature have involved training of the lower limbs and assessments of vascular responsiveness in the upper limb (Watts et al. 2004; Meyer et al. 2006; Tinken et al. 2008; Tjønna et al. 2008; Munk et al. 2009; Guiraud et al. 2012; Bender and Laughlin 2015). This is relevant as, besides the systemic effect of medication, lower limb exercise training exposes the leg vasculature to different hemodynamic forces that might induce different vascular adaptations when compared to the untrained limbs (i.e., differences in vascular responsiveness and endothelial phenotype and function throughout conduit arteries) (Gokce et al. 2002; Hambrecht et al. 2003; Newcomer et al. 2004; Proctor and Newcomer 2006; Laughlin et al. 2008; Bender and Laughlin 2015). Furthermore, microvascular dysfunction is an early complication that leads to changes in cardiovascular hemodynamic function, and ultrasonic assessments of FMD do not provide direct information about microvascular function (Bender and Laughlin 2015). In relation to this, recent studies have shown that the near‐infrared spectroscopy (NIRS) combined with vascular occlusion test (NIRS‐VOT) technique is an in vivo, noninvasive, and reliable tool for assessment of vascular responsiveness within the microvasculature in different populations, limbs and conditions (McLay et al. 2016a,2016b; Soares et al. 2017; Soares et al., 2018b). By using the NIRS‐VOT technique, muscle oxygen saturation (StO_2_) can be measured and microvascular responsiveness can be assessed by calculating the steepness and the area under the curve of the overshoot of the StO_2_ signal during re‐oxygenation.

Thus, considering the importance of assessing the effects of rehabilitation programs on microvascular function in lower limbs, this study aimed to evaluate whether the NIRS‐VOT technique would detect the effects of a 12‐week rehabilitation program including drug administration combined with endurance exercise training on microvascular responsiveness of the leg in a group of coronary heart disease (CHD) patients. We hypothesized that the rehabilitation intervention would increase the steepness of the re‐oxygenation slope and area under the curve of the overshoot of the oxygen saturation signal, indicating improved microvascular responsiveness.

## Methods

### Ethical approval

The study was conducted according to the principles established in the Declaration of Helsinki and was approved by the Human Ethics Committee of University of Flores. Ten patients (7 males and 3 females; 57.3 ± 7.6 years) who presented a variety of documented coronary heart disease (i.e., acute myocardial infarction, coronary artery bypass surgery, transluminal angioplasty with stent, chronic stable angina) and who were taking medications (Table 1) to keep them clinically stable and in sinus rhythm participated in this study. All patients took part in a cardiac rehabilitation program at the Dr. Julio Mendez Hospital (Buenos Aires, Argentina) and had adherence of at least 80% to the exercise training program.

**Table 1:** Medical treatment taken by patients in the study

Medical treatment	Number of patients
Β blockers	10
ACE inhibitors	5
Angiotensin receptor antagonists	3
Antiplatelet agents	10
Statins	10
Others	10

### Maximal incremental ramp test

Before and after the exercise training intervention, all the patients performed a modified Bruce ramp test protocol until exhaustion on a treadmill with handle bars on each side. This test produces a progressive increase of 1 MET per minute by increasing the velocity and inclination of the ergometer. The test started with 2 min standing, a 2‐min warm‐up at 1 km∙h^−1^, and then progressive increments in velocity and inclination every min until exhaustion (i.e., 1.6/0; 2.0/5; 2.7/10; 3.3/10; 3.7/11; 4.0/12; 4.5/12; 5.0/13; 5.4/14; and 6.0/14 km∙h^−1^ and degrees from the first to the last stage).

Heart rate (HR) was measured, beat by beat for the full duration of the test using a 12‐channel electrocardiograph (Cardiovit AT1, Schiller, Switzerland). Maximal heart rate (HR_max_) was the highest HR value observed during the incremental test derived from a 15‐sec rolling average.

### Resting HR and blood pressure

The resting HR was calculated as the average of the last 30 sec during the 2 min prior to the onset of the incremental test. Blood pressure (BP) was measured manually by a trained medical professional, with a sphygmomanometer on the right arm while they kept holding on the handlebar with the left one. Resting systolic blood pressure (SBP) and diastolic blood pressure (DBP) were measured as the average of the last 30 sec before the onset of the incremental exercise.

### Microvascular responsiveness assessment

Microvascular responsiveness was assessed before and after the exercise training intervention as previously described (McLay et al. 2016a; Soares et al. 2017). Briefly, participants laid supine quietly on an examination table for 10 min. Following this resting period, a wireless near‐infrared spectroscopy (NIRS) system (Moxy Muscle Oxygen Sensor, Hutchinson, MN) was placed on the belly of the tibialis anterior muscle, a muscle that has been shown to be susceptible to training related adaptations (Mclay et al. 2016a; Soares et al. 2018a) and located in a region characterized by low subcutaneous adipose tissue layer. As previously described, (Piucco et al. 2018) this device quantifies oxy and deoxyhemoglobin signal in the muscle under the area of NIRS interrogation using a near‐infrared wavelength spectrum light from 670 to 810 nm. The system was consisted of one source of emitter and two detectors separated by 1.25 and 2.5 cm. By measuring changes in light absorption at different wavelengths, changes in oxyhemoglobin ([HbO_2_]) and deoxyhemoglobin ([HHb]) can be measured continuously, and StO_2_ can be calculated (defined as [HbO_2_] / [HbO_2_] + [HHb]). The NIRS system was inserted into a dark rubber shield and then placed on the tibialis anterior muscle, secured in place using double side tape, and loosely wrapped around the site of NIRS probing using an elastic tensor bandage to avoid blood flow constriction while further minimizing movement and light intrusion.

The device stayed attached to the participant for the duration of testing. A cuff that was manually and rapidly inflated (10 sec) for blood flow occlusion was placed below the knee (approximately 5 cm distal to the popliteal fossa). Occlusion pressure was maintained at 250 mmHg for the occlusion time. NIRS measurements were collected continuously at an output frequency of 2 Hz for the entire duration of each test (5 min of baseline, 5 min of occlusion, and 8 min following cuff release). For consistency between visits, the NIRS probe was placed by the same investigator at pre‐ and postintervention and the site of interrogation was identified by asking the patient to bring the tip of the foot up to contract the anterior tibial muscle. Once the muscle was identified, the middle area of the contracted muscle was marked, the subject was asked to relax the foot, and the device was placed right on the mark. This procedure is the same as used in McLay et al. (2016c) and Iannetta et al. (2018) studies, which have been shown to provide repeatable and reliable NIRS measurements of microvascular function.

Microvascular responsiveness was evaluated as previously established (McLay et al. 2016c; Soares et al. 2017). Briefly, baseline oxygen saturation (StO_2_ (%)) was calculated as the average of the last 2 min StO_2_ prior to ischemia. The StO_2_ re‐oxygenation rate was quantified as the upslope of the StO_2_ signal over a 10‐sec period immediately following cuff release (slope 2 StO_2_, %^.^sec^−1^). Over this period, the re‐oxygenation rate represents a linear response which allows for a simple slope calculation. The area under the curve of the StO_2_ overshoot (StO_2AUC_) was calculated as the total area under the curve of percent variation of the StO_2_ signal from the cuff release until the end of the 8 min post‐cuff release. For the StO_2AUC_ calculation, the baseline value represented 0% of variation. The minimal value of the StO_2_signal during occlusion, the maximal value of the StO_2_ signal during re‐oxygenation, and the amplitude of the StO_2_ (Maximum value during re‐oxygenation – minimal value during occlusion) were also calculated. The participants abstained from any medication on the testing day to avoid acute influences of drug on vascular function.

### Exercise training intervention

All the patients performed 12 weeks of mixed training that focused on aerobic performance 2 times per week for 40 min each time. The training consisted of a 12‐min warm‐up and 28 min of high‐intensity interval training (HIIT). HIIT was performed on a treadmill and consisted of four sets of 4 min performed at 90–95% of the maximal heart rate (HR_max_) and 3 min performed at 70% of HR_max_. High‐intensity interval training was chosen based on previous studies showing that this model is feasible and more efficient than moderate continuous exercise in improving cardiovascular function even in elderly patients with chronic heart failure and a range of different cardiovascular complications (Wisloff et al. 2007; Elliott et al. 2015). At the end of the HIIT, participants performed 15 min of light resistance exercise and a 5‐min cool down. The resistance exercise consisted of two sets of four exercises (leg extension, lat pulldown, biceps curl, and triceps pull down) with 8–12 repetitions at 40–60% 1 RM.

### Statistical analysis

Data are presented as mean ± standard deviation. All data were tested for normality using D'Agostino and Pearson normality test. A paired Student's *t*‐test was applied to compare all variables. A *P* value < 0.05 was considered as the level of statistical significance. The effect size for slope 2 and StO_2AUC_ was assessed by performing a Cohen's *d* test. Data analysis was performed using the GraphPad Prism software version 7.0.

## Results

The general characteristics of the patients are described in Table 2. The rehabilitation program increased significantly the aerobic performance from 7.2 ± 1.8 METs at pre‐rehabilitation to 8.3 ± 1.5 METs at post‐rehabilitation and medium effect size (*d *=* *0.66)

**Table 2:** General characteristics of the patients (*n* = 10)

	Pretraining (Mean±SD)	Posttraining (Mean±SD)
Height (cm)	165.1 ± 6.5	165.1 ± 6.5
Body mass (kg)	82.7 ± 14.6	81.8 ± 15.6
BMI (kg/m^2^)	30.3 ± 5.1	30.0 ± 5.4
Blood pressure (mmHg)		
Systolic blood pressure	121.0 ± 7.4	122.0 ± 9.2
Diastolic blood pressure	85.0 ± 5.3	85.5 ± 3.7
Heart rate (bpm)	71.1 ± 9.4	68.9 ± 9.0

Table 3 shows the baseline, minimum, maximum, and amplitude values for the oxygen saturation signal. Although no significant differences were observed between pre‐ and post‐rehabilitation responses for the baseline, minimum, and maximum % StO_2_ (*P* > 0.05), a significant increase in the amplitude of the % StO_2_ was detected after the rehabilitation period (*P* < 0.05). Also, there was a small effect size for baseline (*d *=* *0.26) and minimum (*d *=* *0.43) StO_2_ and large effect size for maximum (*d *=* *1.01) and amplitude (*d *=* *1.03) of StO_2_.

**Table 3:** Profiles of oxygen saturation signal pre‐ and post‐training

	Pre (Mean ± SD)	Post (Mean ± SD)
% StO_2_ baseline	57.9 ± 10.9	55.3 ± 8.5
% StO_2_ minimum	16.5 ± 15.1	10.0 ± 15.0
% StO_2_ maximum	73.5 ± 10.8	82.9 ± 6.9
% StO_2_ amplitude	58.8 ± 17.3	72.9 ± 7.0^a^

Baseline% StO_2_, 2 min average of oxygen saturation signal prior to the occlusion period; Minimum % StO_2_ value, minimal value of the oxygen saturation signal during occlusion; Maximal % StO_2_ value, maximal value of the oxygen saturation signal during reperfusion; % StO_2_ amplitude, difference between minimal and maximal oxygen saturation values.

a Difference between groups (*P* < 0.05); paired Student's *t*‐test.

**Figure 1:**
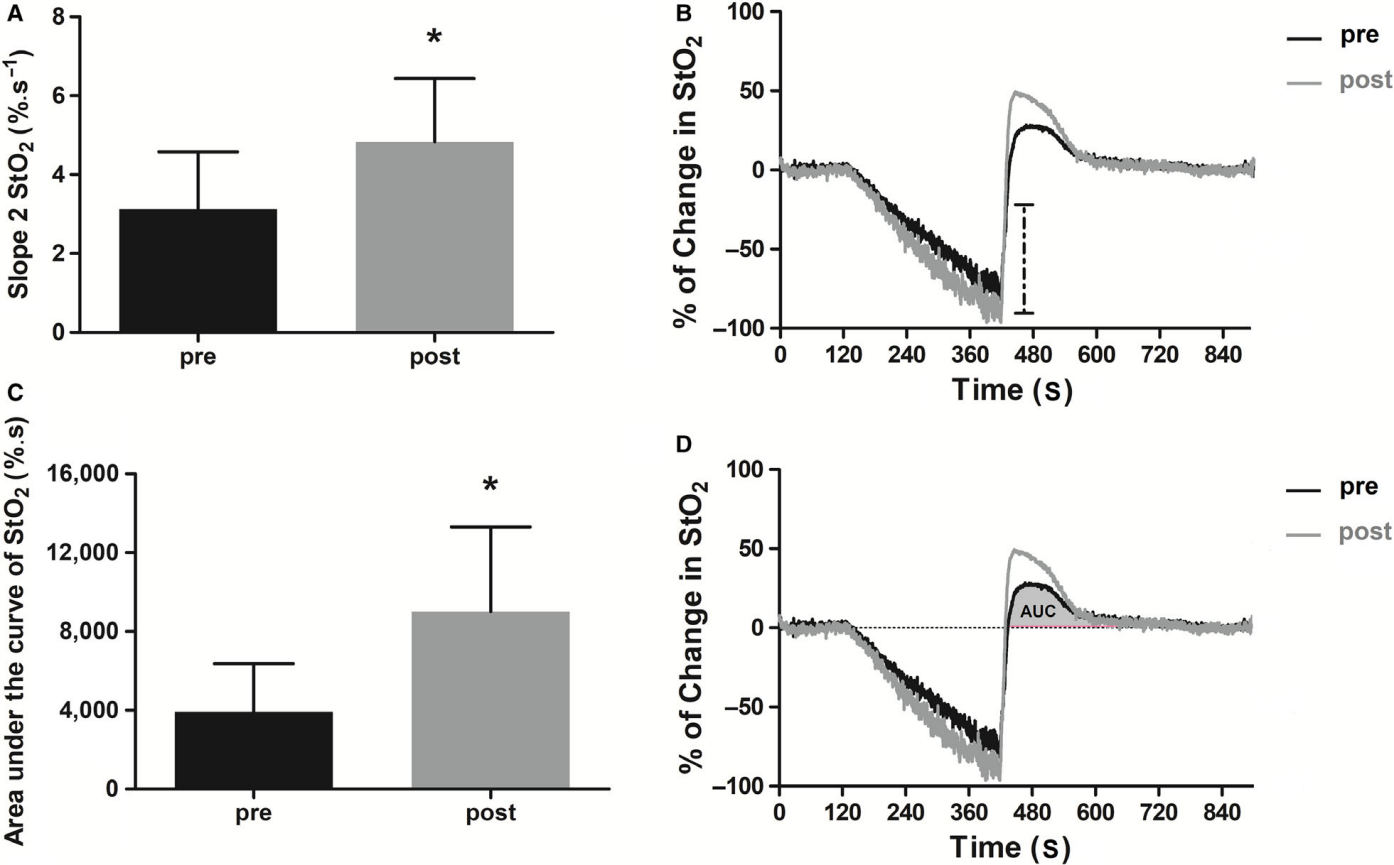
Comparison between pre‐ and post‐training values for Slope 2 of oxygen saturation (Slope 2 StO_2_) and area under the curve of oxygen saturation during reperfusion (StO_2_ _AUC_). Panel A, greater slope 2 StO_2_at post‐rehabilitation compared to the pre‐rehabilitation (mean ± SD); Panel B, representative oxygen saturation profiles highlighting the differences between pre‐ and post‐rehabilitation for the 10‐sec window upslope (slope 2 StO_2_) as indicated by the dashed lines; Panel C, greater StO_2_ _AUC_at post‐rehabilitation compared to the pre‐rehabilitation (mean ± SD); and Panel D, representative profiles highlighting the differences between pre‐ and post‐rehabilitation for area under the curve of StO_2_signal during reperfusion (filled in gray area (AUC)). *Difference between pre‐ and post‐rehabilitation (*P* < 0.05). For comparison between variables, a paired Student's *t‐*test was applied.

The slope 2% StO_2_ was significantly steeper after 12 weeks of intervention (4.8 ± 1.6%∙sec^−1^) compared to the prevalues (3.1 ± 1.6%∙sec^−1^) (*P* < 0.05; Figure 1, panels A and B) and showed a large effect size of 1.06. The area under the curve for the change in the % StO_2_ signal during re‐oxygenation showed a significant increase from 3494 ± 2372%∙sec at pre‐rehabilitation to 9006 ± 4311%∙sec at post‐training (*P* < 0.05; Figure 1, panels C and D) and a large effect size of 1.58.

## Discussion

To the best of our knowledge, this is the first study to investigate whether the NIRS‐VOT technique would detect changes in microvascular responsiveness induced by a 12‐week rehabilitation program that included drug administration combined with endurance training exercise within the lower limb microcirculation in patients with documented coronary heart disease. The main findings from this investigation were that 1) the reperfusion slope (slope 2 StO_2_) increased significantly after the 12 weeks of the rehabilitation intervention, indicating a faster re‐oxygenation rate (i.e., faster vascular responsiveness); and 2) the area under the curve for the StO_2_ signal after cuff release was greater after the rehabilitation program, representing a greater total re‐oxygenation.

The increase in the slope 2 StO_2_ and the greater area under the curve of StO_2_ observed in our study after the intervention have been associated with faster reperfusion and greater vascular responsiveness within the lower limb microcirculation (McLay et al. 2016a,2016b; Soares and Murias 2018). In line with our findings, previous studies investigating the effects of cardiac rehabilitation programs (medication and exercise) on vascular responsiveness in patients with cardiovascular complications have been shown to promote significant enhancements in cardiovascular function in these populations (Gokce et al. 2002; Ades et al. 2011; Currie et al. 2013; Kim et al. 2014; Van Craenenbroeck et al. 2015).

Although positive effects of rehabilitation programs that included medication and exercise training have been reported in the forearm FMD, the vasculature of the lower limb (i.e., the leg) is less explored in the literature. This is important as arms and legs have been shown to be exposed to different hemodynamic stress, thereby undergoing different vascular adaptations and consequently exhibiting different responses to vasoactive substances (Pawelczyk and Levine 1985; Newcomer et al. 2004; Proctor and Newcomer 2006). For instance, Pawelczyk and Levine (1985) showed greater decrease in the calf vascular conductance in response to phenylephrine infusion than in the arm vasculature. Their findings suggested that beta‐adrenergic receptor responsiveness in human limbs is nonuniform and that this greater response to alpha1‐adrenergic receptor stimulation in the lower limb (calf) likely represents the adaptive mechanism related to sustained exposure to higher hemodynamic pressure during standing (Pawelczyk and Levine 1985).

Additionally, although a more systemic effect of drugs administration is expected, prioritizing the assessment of upper limb vascular adaptations to exercise training is surprising given that the vasculature within the trained regions is more likely to be exposed to the chronic adaptations induced by exercise (i.e., expansion of the capillary network, enlargement of conduit arteries (Leung et al. 2008), and overexpression of endothelial proteins associated with increased vascular reactivity (Goto et al. 2003; Rakobowchuk et al. 2008)) than the vascular districts distant from the trained limb (i.e., the arm) (Gokce et al. 2002). These adaptations are associated with better capacity to increase blood flow in response to an ischemia/reperfusion stimuli and larger capillary recruitment (Leung et al. 2008; Phillips et al. 2015). The more prominent effects of exercise training on macrovascular (Gokce et al. 2002; Walther et al. 2008) and microvascular (Soares et al., 2018a) function of the trained limb have already been investigated in healthy individuals and are in agreement with the faster microvascular re‐oxygenation rate in the leg after the exercise training program found in the current study using the NIRS‐VOT technique.

In addition to the faster re‐oxygenation rate, this study showed for the first time increases in the area under the curve of the re‐oxygenation overshoot within the microcirculation, in response to a rehabilitation program including drug administration combined with high‐intensity endurance training exercise in coronary heart disease patients. In relation to this, previous findings from our laboratory (Soares et al. 2017; Soares and Murias 2018; Soares et al., 2018b) have suggested that a larger overshoot of re‐oxygenation occurs in situations where an increase in %FMD is expected.

Among other effects, exercise training improves vascular function by upregulating vascular endothelial nitric oxide synthase enzyme (eNOS) (Leung et al. 2008). Improved NO production results in larger and more sustained dilation of the vasculature when challenged by changes in shear stress (Hambrecht et al. 2003; Leung et al. 2008). Furthermore, chronic adaptations to exercise training are associated with higher levels of antioxidant enzymes, such as superoxide dismutase, catalase, and glutathione peroxidase, which would also facilitate the NO production and improve FMD (Lambertucci et al. 2007). Therefore, it may be inferred that the greater amplitude and area under the curve of the re‐oxygenation overshoot observed in the microvascular assessments in this study are, at least in part, related to higher and more sustained vasodilatory response to shear stress within the microvasculature promoted by the rehabilitation intervention.

### Experimental considerations

Although the beneficial effects of rehabilitation exercise training on the arterial vasculature of CHD patients have been previously described, the rehabilitation programs involving populations with severe cardiovascular disease are usually accompanied by medication, which might also improve cardiovascular health. However, the aim of this study was to show the usefulness of the NIRS‐VOT technique in detecting the changes in lower limb microvascular responsiveness in CHD patients after a 12‐week rehabilitation program independently of the medication or training intervention model. Additionally, although previous studies have assessed the effects of rehabilitation on vascular function, the techniques previously used required specialized personal, expensive equipment and were invasive (infusion of vasoactive substances), thereby reinforcing the advantages of the NIRS‐VOT technique. We also acknowledge that the disease profile of the patient population of the present study is somewhat heterogeneous; thus, further studies with patients affected by a more specific heart disease condition are warranted.

In conclusion, this study showed that the NIRS‐VOT technique is a reliable in vivo and noninvasive technique capable of detecting the improvements in lower limb microvascular responsiveness in individuals with coronary heart disease following a rehabilitation program that included drug administration and endurance exercise training.

## Conflicts of Interest

The authors declare no conflict of interests.
